# Immunological and inflammatory biomarkers of localized and locally advanced prostate cancer treated with radiotherapy

**DOI:** 10.3389/fimmu.2026.1801027

**Published:** 2026-07-28

**Authors:** Kanta Ka, Doudou Georges Massar Niang, Mame Daro Faye, Jaafar Thiam, Paul Sargos, Alberto Bossi, Mario Terlizzi, Mohamed Jalloh, Babacar Sine, Mamadou Moustapha Dieng, Sidy Ka, Babacar Mbengue

**Affiliations:** 1 Radiotherapy Department, Dalal Jamm Hospital, Dakar, Senegal; 2 Service d’Immunologie, Université Cheikh Anta Diop de Dakar, Dakar, Senegal; 3 Division of Radiation Oncology, McGill University Health Centre, Montreal, QC, Canada; 4 Service de Cancérologie, Dalal Jamm Hospital, Dakar, Senegal; 5 Department of Radiotherapy, Institut Bergonié, Bordeaux, France; 6 Department of Radiotherapy, Villejuif, France; 7 Service d’Urologie Hopital General Idrissa Pouye, Dakar, Senegal; 8 Service d’Urologie Centre de Santé de Ngor, Dakar, Senegal; 9 Aristide Le Dantec Hospital, Joliot Curie Institute of Cancer, Dakar, Senegal; 10 Institut Africain de Lutte contre le Cancer, Dakar, Senegal

**Keywords:** biomarkers, inflammation, precision medicine, prostate cancer, radiotherapy, tumor microenvironment

## Abstract

Radiotherapy r5emains a cornerstone of treatment for localized and locally advanced prostate cancer, yet substantial heterogeneity persists in oncological outcomes and treatment-related toxicity. Increasing evidence suggests that systemic inflammation, immune microenvironment characteristics, and tumor-specific molecular features contribute to these variations and may provide clinically relevant biomarkers. This narrative review summarizes current evidence regarding immunological and inflammatory biomarkers in prostate cancer treated with radiotherapy, focusing on their biological rationale, prognostic significance, predictive potential, and clinical applicability. Circulating biomarkers, including neutrophil-to-lymphocyte ratio, platelet-to-lymphocyte ratio, C-reactive protein, fibrinogen, and cytokines such as IL-6 and TGF-β1, have been associated with oncological outcomes and toxicity in several studies. However, their interpretation remains limited by biological nonspecificity, methodological heterogeneity, variable cutoff definitions, and lack of prospective validation. Within the tumor microenvironment, immune cell populations, including CD8^+^ tumor-infiltrating lymphocytes, regulatory T cells, CD163^+^ macrophages, and immune checkpoint molecules, provide insights into tumor–immune interactions and radiotherapy-induced immune modulation. Emerging biomarkers such as extracellular vesicles, circulating tumor DNA, circulating tumor cells, and microRNAs offer promising opportunities for disease monitoring and treatment adaptation, although most evidence remains preliminary, particularly in localized disease. In contrast, genomic classifiers such as Decipher, Prolaris, and OncotypeDX currently represent the most clinically mature biomarker platforms for risk stratification and treatment decision support. Although several biomarkers demonstrate biological plausibility and prognostic value, few have sufficient clinical validation to guide routine radiotherapy decision-making. Future multicenter prospective studies, standardized biomarker assessment protocols, and integrated approaches combining clinical, imaging, molecular, and immune data are required to enable clinically actionable precision radiotherapy strategies.

## 1 Introduction

Prostate cancer is the second most frequently diagnosed malignancy among men worldwide and remains a major cause of cancer-related mortality (1). While radical prostatectomy, androgen deprivation therapy, and chemotherapy are important treatment options, radiotherapy (RT) constitutes a cornerstone modality for patients with localized and locally advanced hormone-sensitive disease (2–4).

Despite continuous improvements in RT techniques, treatment outcomes and toxicity profiles still show substantial heterogeneity. This variability arises in part from complex interactions between tumor biology, immune microenvironment, and systemic inflammatory responses. Tumor hypoxia, cytokine signaling, immune cell infiltration, and stromal remodeling contribute to radioresistance and shape disease trajectories (5, 6).

Unlike other solid tumors, prostate cancer is generally regarded as immunologically “cold,” characterized by low tumor-infiltrating lymphocytes, limited neoantigen expression, and a predominantly immunosuppressive microenvironment (7). This biological context partly explains the challenges in achieving durable immune responses and highlights the importance of identifying and validating biomarkers that may improve prediction of treatment outcomes and toxicity.

Over the past decade, research has increasingly focused on immunological and inflammatory biomarkers to better understand and anticipate treatment outcomes. Circulating cytokines and hematological indices, such as the neutrophil-to-lymphocyte ratio and C-reactive protein, may reflect systemic inflammation and tumor burden. Meanwhile, tumor-associated immune infiltration and checkpoint molecule expression offer insights into local immune dynamics. Emerging blood-based markers, including extracellular vesicles and circulating tumor DNA, further expand the landscape of potential tools to guide precision RT.

This review aims to summarize the current evidence on immunological and inflammatory biomarkers in localized and locally advanced prostate cancer treated with radiotherapy. Particular emphasis is placed on distinguishing biological plausibility, prognostic associations, predictive potential, and clinical utility. We also discuss the current level of validation and readiness for clinical implementation of these biomarkers, as well as their potential role in future precision radiotherapy strategies.

## 2 Materials and methods

### Search strategy for narrative synthesis

A targeted structured literature search was performed in PubMed, Scopus, and Web of Science databases up to October 2025. Search terms included combinations of the following keywords and MeSH terms: “prostate cancer,” “radiotherapy,” “biomarkers,” “immunity,” “inflammation,” “cytokines,” “tumor microenvironment,” “liquid biopsy,” and “circulating tumor DNA.”.

The Boolean query used was:

(“prostate cancer” OR “prostatic neoplasms”) AND (“radiotherapy” OR “radiation therapy”) AND (“biomarker” OR “cytokine” OR “immune” OR “inflammatory marker” OR “tumor microenvironment” OR “liquid biopsy” OR “circulating tumor DNA” OR “microRNA”).

Articles published in English or French were considered. Clinical trials, retrospective and prospective cohort studies, meta-analyses, and relevant reviews were included. Case reports, non-peer-reviewed preprints, and conference abstracts were excluded. Reference lists from relevant publications were screened for additional studies of interest.

Relevant study characteristics, including study design, sample size, biomarker type, treatment setting, clinical endpoints, and main outcomes, were reviewed and summarized to support the narrative synthesis.

As this work was not designed as a formal systematic review, no PRISMA flow diagram, risk-of-bias assessment, or evidence grading framework was applied.

Given the heterogeneity in study designs and biomarker assessment methods, a qualitative synthesis was performed rather than a formal meta-analysis. Effect measures such as hazard ratios, odds ratios, or correlation coefficients were reported when available. Abbreviations were standardized throughout the manuscript following international oncologic and radiotherapy conventions.

## 3 Circulating biomarkers of inflammation and immunity

The analysis of circulating biomarkers in prostate cancer treated with RT provides valuable insights into systemic inflammatory and immune responses associated with irradiation. These biomarkers have been investigated for their potential prognostic and predictive significance, as well as their possible role in treatment personalization. However, the level of clinical validation varies substantially across biomarker categories, and most remain investigational.

### Hematological ratios and acute-phase proteins

Among the most frequently studied circulating biomarkers are the neutrophil-to-lymphocyte ratio (NLR) and the platelet-to-lymphocyte ratio (PLR). The NLR is commonly considered a surrogate marker of systemic immune and inflammatory status. However, both neutrophil and lymphocyte populations are functionally heterogeneous and include subsets with both protumoral and antitumoral activities, making the biological interpretation of NLR complex and context-dependent. In a European cohort of 415 patients treated with radiotherapy, an NLR ≥5 was independently associated with poorer progression-free survival, metastasis-free survival, and overall survival (8). However, these findings have not been consistently reproduced. In a larger Canadian cohort of 1,772 patients, NLR itself was not significantly associated with survival outcomes, whereas an elevated absolute neutrophil count was predictive of overall mortality (9).

Meta-analyses have reported associations between elevated NLR and adverse clinical outcomes in prostate cancer, although substantial heterogeneity exists across studies. For example, a pooled analysis reported that elevated NLR was associated with worse overall survival (HR 1.55, 95% CI 1.37–1.76) and progression-free survival (HR 1.62, 95% CI 1.29–2.04), but not consistently with cancer-specific survival (10, 11). These findings indicate that, although NLR has been associated with systemic disease burden and adverse outcomes, its clinical utility remains limited by variability across patient populations, treatment settings, biomarker cutoffs, and timing of assessment. These discrepancies likely reflect differences in study design, treatment context, and biomarker assessment methods, and highlight the limited specificity of NLR as a standalone biomarker.

Similarly, the platelet-to-lymphocyte ratio (PLR) has been investigated as a complementary inflammatory biomarker. In a meta-analysis including 32 studies and 21,949 patients, elevated pretreatment PLR was associated with poorer overall survival (6 studies; HR 1.72, 95% CI 1.36–2.18), while the association with cancer-specific survival was not statistically significant (3 studies; HR 1.56, 95% CI 0.82–2.97) (12). However, these results were derived from heterogeneous cohorts encompassing various treatment modalities, and data specifically evaluating PLR in radiotherapy-treated prostate cancer remain limited and inconsistent. No standardized cut-off value has been validated in this setting. These findings suggest that PLR currently lacks sufficient robustness for routine clinical use as a prognostic biomarker in radiotherapy-treated populations.

C-reactive protein (CRP) and fibrinogen levels have also been investigated as acute-phase proteins reflecting systemic inflammation and radiotherapy-associated immune modulation. A prospective study reported that radiotherapy was associated with significant increases in NLR, PLR, and fibrinogen levels without significant changes in CRP, suggesting the induction of a chronic low-grade inflammatory state sometimes referred to as “inflammaging” (13). In addition, early elevation of CRP (>5 mg/L) during treatment, combined with a decline in hemoglobin, was associated with increased risk of acute genitourinary toxicity, whereas higher lymphocyte counts appeared protective against late genitourinary and gastrointestinal toxicities (14). However, variability in study size, timing of measurement, treatment characteristics, and patient-related factors limits the reproducibility and clinical applicability of these findings.

The heterogeneity observed across studies likely reflects differences in clinical and treatment-related contexts rather than purely inconsistent biomarker performance. The prognostic significance of inflammatory markers such as NLR, PLR, and CRP may vary according to the radiotherapy setting (definitive versus postoperative adjuvant or salvage radiotherapy), treatment delivery (including fractionation schemes and irradiated volumes), the use and duration of androgen deprivation therapy, and patient-related factors such as age, comorbidities, smoking status, obesity, concomitant medications, and other conditions known to influence systemic inflammatory indices. These variables should be taken into account when interpreting available evidence, as they may partly explain the variability of reported associations.

Overall, although NLR, PLR, CRP, and fibrinogen are biologically plausible biomarkers and have demonstrated prognostic associations in several studies, they currently lack sufficient analytical standardization and prospective validation to guide routine radiotherapy decision-making. A summary of key hematological biomarkers, their biological rationale, and their reported clinical associations in prostate cancer treated with radiotherapy is presented in **Table 1**.

**Table 1:** Key circulating biomarkers studied in prostate cancer treated with radiotherapy: prognostic and predictive value.

Biomarker	Type	Immunological/inflammatory role	Clinical association (prognosis/toxicity)	References
NLR (Neutrophil-to-Lymphocyte Ratio)	Hematological ratio	Surrogate marker of systemic inflammation and immune status	Elevated NLR has been associated with worse overall survival and shorter PFS in some cohorts; results vary across disease stages and treatment settings	(8)
PLR (Platelet-to-Lymphocyte Ratio)	Hematological ratio	Marker of systemic inflammation and platelet activation	Elevated pretreatment PLR has been associated with poorer overall survival (HR 1.72, 95% CI 1.36–2.18); no significant association with cancer-specific survival (HR 1.56, 95% CI 0.82–2.97)	(12)
CRP (C-reactive protein)	Acute-phase protein	Marker of systemic inflammatory response	Early CRP >5 mg/L has been associated with acute GU toxicity; prognostic value remains inconsistent across studies	(14)
IL-6	Pro-inflammatory cytokine	Pleiotropic cytokine involved in inflammation and STAT3 signaling	Higher IL-6 levels have been associated with GU toxicity and fatigue in limited prospective studies	(15, 16)
TGF-β1	Immunosuppressive cytokine	Involved in fibrosis, tissue remodeling, and immune regulation	Elevated TGF-β1 levels have been associated with ≥ grade 2 late GU toxicity in limited studies	(17)
Exosomal miR-21/miR-375	Exosome-derived microRNA	Candidate regulators of tumor progression and treatment resistance	Associated with adverse prognosis and tumor progression in exploratory studies	(18)
ctDNA (AR, BRCA2, TP53)	Circulating tumor DNA	Tumor-specific genomic alterations	Associated with biochemical relapse, treatment resistance, and adverse prognosis, primarily in advanced disease	(19, 20)

### Cytokines and pro-inflammatory mediators

Cytokines play a central role in the immune landscape of prostate cancer, acting as mediators of both antitumor immunity and tumor-associated immunosuppression. Key immunosuppressive cytokines, such as transforming growth factor-beta (TGF-β) and interleukin-10 (IL-10), promote regulatory T-cell (Treg) differentiation and suppress antitumor immune responses, thereby facilitating myeloid-derived suppressor cell (MDSC) expansion and reinforcing tumor-associated immune evasion (21, 22). Conversely, pro-inflammatory cytokines such as interferon-gamma (IFN-γ), tumor necrosis factor-alpha (TNF-α), interleukin-1 (IL-1), and interleukin-2 (IL-2), which may be produced by activated immune cell populations including M1-like macrophages, have been associated with antitumor immune activity (23).

Radiotherapy has been shown to modulate cytokine signaling pathways in a dose- and context-dependent manner. Low radiation doses have been reported to increase IL-2 and IFN-γ production, potentially enhancing natural killer (NK) cell activity, whereas higher doses may reduce IL-12 secretion and alter immune cell function (24). These observations underscore the complexity of cytokine networks and the context-dependent nature of their biological effects. Cytokine profiling, including interferons, interleukins, and TNF-family mediators, has therefore been proposed as a potential approach for characterizing immune responses and identifying candidate biomarkers for treatment monitoring (25). However, substantial variability across studies, including differences in assay platforms, sample processing, timing of collection, and cohort characteristics, limits the reproducibility and clinical interpretation of these findings. Representative cytokines, their biological functions, and reported clinical associations are summarized in **Table 2**.

**Table 2:** Key circulating cytokines and immune biomarkers in prostate cancer treated with radiotherapy: prognostic and predictive implications.

Biomarker	Type	Biological role	Clinical association (prognosis/predictive)	References
CD8^+^ Tumor-Infiltrating Lymphocytes (TILs)	Local immune marker	Cytotoxic T-cell infiltration; antitumor immune response	Higher CD8^+^ TIL density has been associated with improved oncological outcomes, primarily in prostatectomy cohorts; evidence in RT-treated patients remains limited	(21, 26)
Regulatory T cells (Tregs) and CD163^+^ macrophages	Local immune marker	Immunosuppressive tumor microenvironment	Increased Tregs and CD163^+^ macrophages have been associated with adverse outcomes and disease progression in exploratory studies	(27)
PD-L1/PD-L2	Immune checkpoint molecules	Mediators of adaptive immune resistance	PD-L1 expression may increase following RT; higher PD-L2 expression has been associated with shorter metastasis-free survival and increased biochemical recurrence	(28, 29)
Tertiary Lymphoid Structures (TLS)	Organized immune aggregates	Local antigen presentation and adaptive immune activation	Presence of TLS has been associated with favorable immune responses in several cancers; role in RT-treated prostate cancer remains under investigation	(30)
Decipher Genomic Classifier	22-gene RNA expression panel	Risk stratification of aggressive disease	Associated with risk of distant metastasis and adverse oncological outcomes; may support adjuvant or salvage RT decision-making	(31)
Prolaris (Cell Cycle Progression score)	Gene expression panel	Assessment of tumor proliferative activity	Associated with biochemical recurrence and disease-specific mortality risk, particularly in low- and intermediate-risk disease	(32)
OncotypeDX Genomic Prostate Score (GPS)	17-gene panel	Molecular risk stratification	Associated with adverse pathology and recurrence risk; may assist treatment selection in localized disease	(33)
GSTP1, APC, RASSF1 methylation	Epigenetic biomarkers	Epigenetic alterations associated with tumor progression	Aberrant methylation has been associated with aggressive pathological features and recurrence risk; clinical utility remains investigational	(34)

The interpretation of cytokine dynamics in prostate cancer is highly dependent on treatment context. Variations in radiotherapy dose and fractionation, irradiated volumes, concomitant androgen deprivation therapy, baseline patient characteristics, and timing of sample collection may all significantly influence cytokine levels and their association with clinical outcomes, thereby contributing to the heterogeneity observed across studies.

Several cytokines have been investigated as potential biomarkers of radiotherapy-related toxicity. Prospective studies have reported associations between increased IL-6 and IL-2 levels during treatment and a higher incidence of acute genitourinary toxicity (15). Elevated pretreatment TGF-β1 levels have also been associated with an increased risk of ≥ grade 2 late genitourinary toxicity, consistent with its established role in fibrosis and tissue remodeling (17). Similarly, higher baseline IL-6 concentrations have been correlated with increased fatigue scores during treatment, while elevated TGF-β1 levels before and during radiotherapy have been associated with higher genitourinary toxicity grades (16). Nevertheless, these observations originate from a limited number of studies, often involving relatively small cohorts, and should therefore be interpreted cautiously.

Beyond toxicity, exploratory studies have suggested potential associations between cytokine dynamics and treatment efficacy. Elevated IL-6 and IL-8 levels have been associated with markers of disease activity, including higher PSA levels, whereas increased IFN-γ production following irradiation has been linked to enhanced antitumor immune activity (24, 35). However, these findings remain preliminary and do not establish a validated predictive role for these cytokines in radiotherapy-treated prostate cancer.

Overall, cytokines such as IL-6, IL-2, IL-8, IFN-γ, and TGF-β1 are biologically plausible and clinically interesting biomarkers. Nevertheless, their analytical validity, reproducibility, optimal sampling conditions, and clinical utility remain insufficiently established. At present, cytokine-based biomarkers should be considered investigational, and larger prospective studies with standardized methodologies are required before their incorporation into routine radiotherapy decision-making.

## 4 Tumor microenvironment and immunogenicity

The prostate tumor microenvironment is generally characterized by low immunogenicity, with limited lymphocyte infiltration, relatively low tumor mutational burden, and modest baseline expression of immune checkpoint molecules (36). These features contribute to the classification of prostate cancer as an immunologically “cold” tumor. Nevertheless, radiotherapy can induce significant alterations in the local immune milieu, modifying cellular interactions within the tumor microenvironment and potentially influencing treatment response.

Intratumoral infiltration by lymphocytes, particularly CD8^+^ T cells, has been widely investigated as a potential biomarker of antitumor immunity. Several studies have reported associations between higher CD8^+^ T-cell densities and improved oncological outcomes, primarily in prostatectomy cohorts (26). However, evidence supporting a similar prognostic or predictive role in radiotherapy-treated patients remains limited. Consequently, findings derived from surgical specimens should not be directly extrapolated to patients receiving definitive or salvage radiotherapy. In contrast, increased infiltration by regulatory T cells (Tregs) and CD163^+^ macrophages, often considered markers of an immunosuppressive phenotype, has been associated with less favorable clinical outcomes and an increased risk of disease progression in some studies (27). Collectively, these observations emphasize the complexity of the prostate tumor microenvironment and the need for radiotherapy-specific validation of immune biomarkers.

Radiotherapy, particularly hypofractionated approaches such as stereotactic body radiotherapy (SBRT), may induce dynamic and sometimes paradoxical changes within the tumor microenvironment. Some studies have reported transient reductions in CD8^+^ T-cell infiltration accompanied by increased infiltration of CD163^+^ macrophages following irradiation, suggesting a complex interplay between immune activation and adaptive immunosuppressive mechanisms (27). These findings support the concept that radiotherapy can modulate tumor immunogenicity; however, the extent to which radiotherapy consistently converts prostate cancer from a “cold” to a more inflamed phenotype remains uncertain and likely depends on factors such as radiation dose, fractionation, tumor burden, and treatment sequencing.

Among immune checkpoint pathways, the PD-1/PD-L1 axis has been the most extensively studied. Experimental and translational studies suggest that PD-L1 expression may increase following irradiation, potentially representing an adaptive resistance mechanism triggered by treatment-induced immune activation (28). Beyond PD-L1, PD-L2 has also emerged as a potential biomarker of interest. In a genomic analysis, higher PD-L2 expression was associated with shorter metastasis-free survival and increased risk of biochemical recurrence (29). Nevertheless, the prognostic and predictive significance of checkpoint-related biomarkers remains inconsistent across studies because of variations in patient populations, treatment settings, assay methodologies, and clinical endpoints. At present, neither PD-L1 nor PD-L2 has been validated for routine clinical decision-making in localized prostate cancer treated with radiotherapy.

Tertiary lymphoid structures (TLS) are organized aggregates of B cells, T cells, and dendritic cells that facilitate local antigen presentation and adaptive immune responses (30). Although their role in prostate cancer remains incompletely understood, emerging evidence suggests that radiotherapy may promote TLS formation and enhance local immune activation. These observations have generated interest in combining radiotherapy with immunomodulatory strategies, although the clinical significance of TLS induction in localized disease remains to be established.

More broadly, the limited efficacy of immune checkpoint inhibitors in unselected prostate cancer populations is thought to reflect the highly immunosuppressive nature of the tumor microenvironment, characterized by low T-cell infiltration, enrichment of suppressive immune populations, and expression of inhibitory molecules such as B7-H3 (7). Radiotherapy has therefore been proposed as a potential strategy to enhance tumor immunogenicity and improve responsiveness to immunotherapy. However, clinical evidence supporting this paradigm in localized and locally advanced prostate cancer remains limited. While biologically plausible, the hypothesis that radiotherapy can reliably sensitize prostate tumors to immune checkpoint blockade has not yet been conclusively demonstrated and should currently be considered investigational.

**Figure 1:**
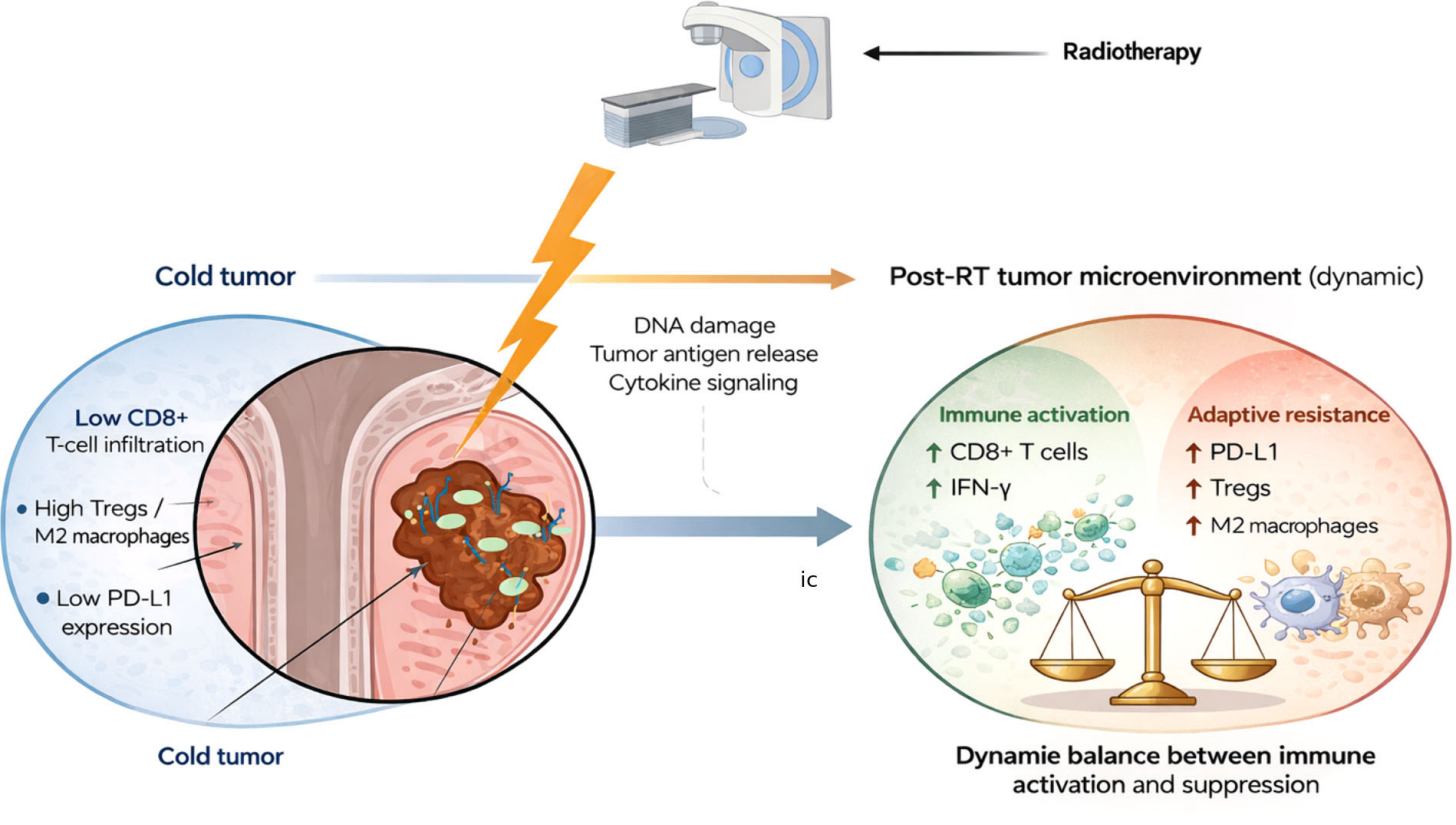
Radiotherapy-induced modulation of the tumor microenvironment in prostate cancer. Prostate tumors are typically immunologically “cold.” characterized by low CD8+T-cell infiltration and an immunonosuppressive microenvionment. Radiotherapy induces DNA damage and tumor antigen release, leading to immune activation and increased cytotoxic T-cell infiltration. At the same time, adaptive resistance mechanisms, including PD-L1. upregulation and expansion of immunosuppressive cell populations such as regulatory T-cells and M2 macrophages, may counterbalance this effect. The resulting immune response is theretore.

Overall, biomarkers derived from the tumor microenvironment provide important biological insights into host-tumor interactions and treatment response. However, most remain at the stage of clinical validity rather than clinical utility, and further prospective radiotherapy-specific studies are required before their implementation in routine practice. The radiotherapy effects in prostate cancer microenvironement are summarized in **Figure 1**.

## 5 Emerging molecular biomarkers

Beyond conventional inflammatory biomarkers, increasing attention has focused on emerging molecular biomarkers capable of providing a more dynamic and tumor-specific assessment of disease biology. These approaches, often grouped under the concept of liquid biopsy, include extracellular vesicles (EVs), circulating tumor DNA (ctDNA), circulating tumor cells (CTCs), and circulating microRNAs. Although these biomarkers offer considerable biological and translational potential, most remain investigational in localized and locally advanced prostate cancer treated with radiotherapy.

### Extracellular vesicles and circulating nucleic acids

Extracellular vesicles are membrane-bound particles secreted by both tumor and stromal cells that contain proteins, DNA, RNA, and other bioactive molecules involved in intercellular communication. Experimental studies suggest that EVs may contribute to immune modulation, tumor progression, and radioresistance in prostate cancer (37). Consequently, EVs have attracted interest as potential biomarkers for disease characterization, treatment monitoring, and early detection of recurrence.

However, despite their biological plausibility, clinical translation remains limited. Significant variability exists in EV isolation techniques, characterization methods, and cargo analysis, resulting in substantial inter-study heterogeneity and limited reproducibility (38, 39). At present, EV-based biomarkers demonstrate biological relevance and emerging clinical validity, but their analytical validity and clinical utility remain insufficiently established for routine implementation.

Circulating microRNAs, either encapsulated within EVs or freely circulating in plasma, have also been investigated as potential biomarkers. Molecules such as miR-21 and miR-375 have been associated with disease aggressiveness and adverse outcomes, while detection of AR-V7 transcripts in exosomal RNA has been linked to resistance to androgen receptor-targeted therapies (18). Nevertheless, most available data originate from advanced disease settings, and prospective validation in radiotherapy-treated localized disease remains limited.

### ctDNA and CTCs

Circulating tumor DNA provides a non-invasive means of detecting tumor-specific genomic alterations and monitoring disease evolution over time. Several studies have demonstrated associations between ctDNA dynamics and treatment response, emergence of resistant clones, biochemical recurrence, and survival outcomes (19, 20). In advanced prostate cancer, alterations involving AR, BRCA2, TP53, and other driver genes have been associated with treatment resistance and adverse prognosis (18).

However, the clinical relevance of ctDNA differs substantially according to disease stage. In metastatic prostate cancer, ctDNA has demonstrated both clinical validity and growing clinical utility. In contrast, localized and locally advanced prostate cancers typically release very low quantities of tumor DNA into the circulation, limiting assay sensitivity and reducing detection rates. Consequently, although ctDNA is biologically attractive for monitoring radiotherapy-treated patients, its role in localized disease remains investigational and requires further prospective validation.

Comprehensive reviews have described the main analytical platforms used for ctDNA assessment, including droplet digital PCR, BEAMing, targeted amplicon sequencing, and next-generation sequencing (40). Despite continuous technological advances, challenges related to sensitivity, specificity, cost, and assay standardization remain major barriers to widespread clinical adoption (41).

Circulating tumor cells represent another source of minimally invasive molecular information. Enumeration of CTCs has demonstrated prognostic value in advanced prostate cancer, while molecular characterization, including androgen receptor splice variant analysis, may provide predictive information regarding systemic therapy response. However, similar to ctDNA, CTC detection remains technically challenging in localized disease because of their low abundance. Their role in patients receiving definitive radiotherapy therefore remains largely exploratory.

### Genomic and epigenomic signatures

Compared with inflammatory and immune biomarkers, genomic classifiers currently represent the most clinically mature biomarker category in localized prostate cancer. Commercial assays such as Decipher, Prolaris, and OncotypeDX Genomic Prostate Score (GPS) integrate the expression of genes involved in tumor proliferation, differentiation, and biological aggressiveness to refine risk stratification beyond conventional clinicopathological factors (31–33).

Importantly, these assays are primarily prognostic rather than predictive biomarkers. Multiple retrospective and prospective validation studies have demonstrated their ability to predict biochemical recurrence, metastasis, and prostate cancer-specific mortality (31, 32). For example, the Decipher genomic classifier has consistently identified patients at higher risk of distant metastasis and adverse oncological outcomes after surgery or radiotherapy and may help inform decisions regarding treatment intensification or postoperative therapy (31).

Similarly, Prolaris has demonstrated prognostic value for biochemical recurrence and disease-specific mortality, particularly among patients with low- and intermediate-risk disease (32). The OncotypeDX GPS assay has been associated with adverse pathology and recurrence risk and may assist in selecting candidates for active surveillance or treatment escalation (33).

Although these genomic classifiers have demonstrated clinical validity and have achieved limited clinical implementation in selected settings, evidence supporting a specific predictive effect for radiotherapy benefit, immune response, or radiosensitivity remains less robust. Their role should therefore be interpreted primarily as risk stratification tools rather than definitive predictors of radiotherapy efficacy.

Beyond transcriptomic classifiers, epigenetic biomarkers have also emerged as potential prognostic tools. Aberrant methylation of genes such as GSTP1, APC, and RASSF1 has been associated with aggressive pathological features and biochemical recurrence (34). Similarly, emerging histone modification signatures may provide additional biological information regarding tumor behavior. However, these biomarkers remain at an early stage of development, and their analytical validity, clinical utility, and cost-effectiveness require confirmation in prospective radiotherapy-specific cohorts before routine adoption can be recommended.

Overall, while liquid biopsy technologies and molecular classifiers offer substantial promise for precision oncology, their level of evidence and clinical readiness varies considerably. At present, genomic classifiers represent the most mature tools for clinical use, whereas EVs, ctDNA, CTCs, microRNAs, and most epigenetic biomarkers should still be considered investigational in localized and locally advanced prostate cancer treated with radiotherapy.

## 6 Clinical perspectives and future integration

### Prediction of efficacy versus toxicity

A major objective of biomarker research in prostate cancer is to improve prediction of both treatment efficacy and treatment-related toxicity. However, it is important to distinguish between biomarkers that are biologically plausible, those that demonstrate prognostic associations, and those that possess sufficient evidence to guide clinical decision-making.

Systemic inflammatory markers such as the neutrophil-to-lymphocyte ratio (NLR), platelet-to-lymphocyte ratio (PLR), C-reactive protein (CRP), and circulating cytokines have repeatedly been associated with oncological outcomes and toxicity following radiotherapy (15, 17, 42). Nevertheless, these biomarkers remain highly nonspecific and are influenced by numerous confounding factors, including age, obesity, smoking, metabolic syndrome, inflammatory diseases, concomitant medications, androgen deprivation therapy, irradiated volumes, fractionation schedules, and timing of blood sampling. Consequently, although these markers demonstrate biological plausibility and some degree of clinical validity, they currently lack sufficient reproducibility and standardization for routine radiotherapy decision-making.

Similarly, local immune biomarkers such as tumor-infiltrating lymphocytes, CD163^+^ macrophages, PD-L1, and PD-L2 provide important insights into tumor-host interactions and may contribute to prognostic assessment (26–29). However, most available evidence derives from retrospective analyses or surgical cohorts, and radiotherapy-specific validation remains limited. Their predictive value for radiotherapy response or toxicity therefore remains uncertain.

Emerging predictive models combining clinical variables, imaging biomarkers, radiomics, genomic classifiers, and immune parameters have demonstrated encouraging performance in retrospective studies (43). Such multimodal approaches may ultimately provide greater predictive accuracy than any individual biomarker. However, prospective multicenter validation and standardized biomarker assessment remain necessary before clinical implementation.

### Ethnic specificity and biomarker generalizability

Increasing evidence suggests that biomarker performance may vary across populations with different genetic ancestries. Men of African ancestry experience disproportionately higher prostate cancer incidence, more aggressive disease presentation, and higher mortality rates than many other populations (44, 45).

Molecular studies have identified differences in genomic alterations, including a lower prevalence of TMPRSS2–ERG fusions and distinct immune and inflammatory profiles in tumors from African populations (44, 45). Variations in systemic inflammatory markers, including CRP and NLR, have also been reported. These observations raise the possibility that biomarker thresholds, predictive performance, and clinical utility may differ between populations.

Despite this, most biomarker studies have been conducted in European or North American cohorts. As a result, the external validity of many proposed biomarkers remains uncertain in underrepresented populations. Future biomarker development should therefore prioritize inclusion of diverse ancestries in clinical trials, genomic datasets, and biobanks to ensure equitable implementation of precision oncology approaches worldwide.

### Precision medicine and adaptive radiotherapy

The development of liquid biopsy technologies has generated considerable interest in adaptive treatment strategies based on dynamic monitoring of tumor biology. Circulating tumor DNA, circulating tumor cells, extracellular vesicles, and circulating microRNAs may provide longitudinal information regarding treatment response, minimal residual disease, and emergence of resistant clones (40, 41).

However, the maturity of these technologies varies considerably. While ctDNA and CTC analyses have demonstrated clinical validity in advanced prostate cancer, their sensitivity remains limited in localized disease because of the low quantity of circulating tumor material. Consequently, their routine application in localized and locally advanced prostate cancer treated with radiotherapy remains investigational.

By contrast, genomic classifiers such as Decipher, Prolaris, and OncotypeDX Genomic Prostate Score currently represent the most clinically mature biomarkers available in localized prostate cancer (31–33). These assays have demonstrated reproducible prognostic value and have achieved limited integration into clinical practice. Nevertheless, their role remains primarily prognostic rather than predictive, and evidence supporting radiotherapy-specific treatment selection remains less robust.

Future precision radiotherapy strategies will likely require integration of multiple complementary data sources, including clinical variables, genomic classifiers, liquid biopsy analyses, imaging biomarkers, and immune profiling. Combining these approaches may improve prediction of recurrence, toxicity, and treatment response while reducing both overtreatment and undertreatment.

### Clinical readiness and current limitations

Despite substantial progress in biomarker research, very few immune or inflammatory biomarkers are currently ready for routine clinical use in patients receiving radiotherapy for localized or locally advanced prostate cancer.

Most biomarkers discussed in this review remain at the stage of biological plausibility or clinical validity rather than demonstrated clinical utility. Methodological heterogeneity, variability in assay platforms, lack of standardized thresholds, inconsistent timing of sample collection, and limited prospective validation continue to hinder clinical translation (40, 41).

Furthermore, many available studies remain retrospective, single-center, and relatively small, limiting generalizability. Even biomarkers that consistently demonstrate prognostic associations, such as NLR, PLR, CRP, and selected cytokines, have not yet been prospectively validated as tools capable of modifying treatment decisions and improving patient outcomes.

Practical considerations must also be addressed before implementation, including cost-effectiveness, regulatory approval, laboratory standardization, data management, and patient acceptance. Consequently, widespread adoption of biomarker-guided radiotherapy strategies cannot currently be recommended outside selected clinical contexts.

Overall, genomic classifiers currently represent the most clinically mature biomarker category, whereas inflammatory markers, cytokines, liquid biopsy approaches, and most tumor microenvironment biomarkers should still be considered investigational. Large prospective multicenter studies incorporating diverse populations and harmonized methodologies will be essential to determine which biomarkers can ultimately achieve sufficient clinical utility to support personalized radiotherapy in prostate cancer.

## 7 Pathomic and multimodal AI-based biomarker integration

Recent advances in pathomics, radiomics, and artificial intelligence (AI)-driven multimodal data integration have expanded the landscape of biomarker discovery in prostate cancer. Pathomics enables the extraction of quantitative features from digitized histopathology slides, whereas radiomics captures imaging-derived phenotypes from modalities such as multiparametric MRI and PSMA PET. Integrating these data with genomic, immunological, and clinical information may provide a more comprehensive representation of tumor biology than any individual biomarker alone.

Several multimodal AI approaches have been developed to support clinically relevant decisions in patients receiving radiotherapy. Proposed applications include treatment intensification or de-escalation, selection of pelvic nodal irradiation, optimization of androgen deprivation therapy (ADT) duration, prediction of biochemical recurrence, identification of patients at risk for treatment-related toxicity, and refinement of active surveillance eligibility. These approaches increasingly integrate histopathological features, including adverse histological variants associated with poorer pathological and oncological outcomes (47), together with imaging, genomic, and clinical variables. In multiple retrospective studies, multimodal models combining these data have demonstrated improved predictive performance compared with conventional clinicopathological factors alone (46, 48).

However, the maturity of these AI-derived biomarkers varies considerably. Most currently available models have been developed and tested using retrospective datasets, often originating from single institutions or highly curated research cohorts. Although some studies have reported external validation in independent datasets, prospective validation remains uncommon. More importantly, few studies have demonstrated true clinical utility, defined as improved patient outcomes or improved treatment decision-making when AI-derived predictions are incorporated into clinical practice.

These approaches are closely related to the emerging concept of digital-twin radiotherapy, whereby machine-learning models attempt to simulate individual tumor behavior and treatment response through integration of multimodal data. While conceptually attractive, digital-twin strategies remain largely exploratory and have not yet been prospectively validated in localized or locally advanced prostate cancer.

Several barriers continue to limit clinical implementation. These include the need for large, diverse, and well-annotated datasets; standardization of image acquisition and feature extraction; reproducibility across institutions and platforms; model interpretability and explainability; and regulatory approval. Furthermore, algorithm performance may vary across patient populations, raising concerns regarding generalizability and potential amplification of existing healthcare disparities.

Consequently, AI-based biomarkers currently demonstrate strong biological plausibility and encouraging early clinical validity, but evidence supporting routine clinical use remains limited. Future prospective multicenter studies will be required to establish whether multimodal AI models can provide sufficient clinical utility to support individualized radiotherapy decision-making in prostate cancer. At present, these technologies should be viewed as promising complementary tools for risk stratification and hypothesis generation rather than established components of routine clinical practice.

## Conclusion

Immunological and inflammatory biomarkers provide valuable insights into treatment response, toxicity, and tumor-host interactions in localized and locally advanced prostate cancer treated with radiotherapy. Circulating inflammatory markers, cytokines, tumor microenvironment features, liquid biopsy approaches, and genomic classifiers each contribute complementary biological information, but their levels of evidence and clinical maturity vary considerably.

While several biomarkers have demonstrated biological plausibility and clinical validity, few are currently ready for routine clinical implementation. Inflammatory markers and most immune biomarkers remain limited by methodological heterogeneity, lack of standardization, and insufficient prospective validation. In contrast, genomic classifiers such as Decipher, Prolaris, and OncotypeDX currently represent the most clinically mature biomarker category, although their role remains primarily prognostic.

Future progress will require prospective multicenter studies, standardized biomarker assessment protocols, and integration of molecular, immune, imaging, and artificial intelligence-derived data. Demonstrating not only clinical validity but also clinical utility will be essential to achieve truly personalized, immune-informed radiotherapy for prostate cancer.
